# Biomimetic delivery of signals for bone tissue engineering

**DOI:** 10.1038/s41413-018-0025-8

**Published:** 2018-08-29

**Authors:** Ming Dang, Laura Saunders, Xufeng Niu, Yubo Fan, Peter X. Ma

**Affiliations:** 1 Macromolecular Science and Engineering Center, University of Michigan, Ann Arbor, MI USA; 2 Key Laboratory for Biomechanics and Mechanobiology of Ministry of Education, School of Biological Science and Medical Engineering, Beihang University, Beijing, China; 3 Beijing Advanced Innovation Center for Biomedical Engineering, Beihang University, Beijing, China; 4 Department of Biologic and Materials Sciences, University of Michigan, Ann Arbor, MI USA; 5 Department of Biomedical Engineering, University of Michigan, Ann Arbor, MI USA; 6 Department of Materials Science and Engineering, University of Michigan, Ann Arbor, MI USA

## Abstract

Bone tissue engineering is an exciting approach to directly repair bone defects or engineer bone tissue for transplantation. Biomaterials play a pivotal role in providing a template and extracellular environment to support regenerative cells and promote tissue regeneration. A variety of signaling cues have been identified to regulate cellular activity, tissue development, and the healing process. Numerous studies and trials have shown the promise of tissue engineering, but successful translations of bone tissue engineering research into clinical applications have been limited, due in part to a lack of optimal delivery systems for these signals. Biomedical engineers are therefore highly motivated to develop biomimetic drug delivery systems, which benefit from mimicking signaling molecule release or presentation by the native extracellular matrix during development or the natural healing process. Engineered biomimetic drug delivery systems aim to provide control over the location, timing, and release kinetics of the signal molecules according to the drug’s physiochemical properties and specific biological mechanisms. This article reviews biomimetic strategies in signaling delivery for bone tissue engineering, with a focus on delivery systems rather than specific molecules. Both fundamental considerations and specific design strategies are discussed with examples of recent research progress, demonstrating the significance and potential of biomimetic delivery systems for bone tissue engineering.

## Biomechanics: Drug delivery systems in bone tissue engineering

Bone tissue engineering offers exciting possibilities for repairing bone defects or regenerating bone tissue for transplantation, and biomimetic drug delivery systems (DDSs) hold promise in providing an environment to support the tissue regeneration. Biomimetic DDSs mimic the release of signaling molecules during development or in the natural healing process, and a team headed by Peter Ma at the University of Michigan in the United States conducted a review of the biomimetic strategies that have been adopted for bone tissue engineering. Various DDSs have been developed that mimic the natural healing or development processes, and they provide controlled drug release. The authors conclude that integrating DDSs with bone implants or scaffolds (which stimulate the growth of bone cells on their surface) could lead to advanced tissue engineering therapy for repairing bone defects.

## Introduction

Bone loss or damage can result from various causes, including degenerative diseases, surgery, and trauma, significantly compromising patient quality of life.^1,2^ Bone possesses an intrinsic ability to repair itself, but there are many situations where complete bone regeneration cannot occur and needs to be stimulated.^3^ Millions of patients suffering from bone defects require bone grafts or substitutes. The market of bone grafts and substitutes was valued at over 2.3 billion US dollars in 2015 and is expected to reach over 3.6 billion US dollars between 2016 and 2024.^4^ Currently, the standard bone-grafting approaches used in clinical practice are autologous, allogeneic, or xenogeneic bone grafts.^5,6^ Autologous bone grafting is considered the gold standard treatment, in which host bone is removed from another site to fill the defect. However, many limitations remain, including multiple required surgeries, potential morbidity, and the limited quantity of donor tissues.^7–9^ Allogeneic or xenogeneic grafts, the transplantation of bone tissue from another human donor or other species, are optional treatments but have inherent limitations including immunogenic responses, infection, and pathogen transmission risks.^10–13^ For these reasons, bone tissue engineering has emerged as a potential new therapy. The tissue engineering approach utilizes biomaterials to replicate the microenvironment and stimulate bone regeneration.^14,15^ In general, a tissue engineering system involves three components: the scaffold, cells, and signaling cues. An engineered scaffold can serve as a temporary extracellular matrix (ECM) to support three-dimensional bone tissue regeneration. It is often beneficial for the tissue-engineering scaffold to mimic or replicate certain advantageous features of the natural ECM, while incorporating some artificially engineered features could be advantageous in stimulating and accelerating the regeneration process.

A tissue-engineering scaffold is critical to provide the microenvironment for regenerative cells, supporting cell attachment, proliferation, differentiation, and new tissue synthesis.^14,16^ Biomimetic approaches to the replication of the features of the native ECM in a bone tissue engineering scaffold incorporate many design features including material composition,^17,18^ biodegradability and mechanical properties,^19,20^ architectural topology,^21,22^ surface features,^23,24^ etc. Innovative technologies in chemistry and processing have been developed to achieve biomimetic scaffolding capable of mimicking the native ECM on various levels. More detailed discussion and comprehensive reviews of biomimetic scaffolds can be found in the literature.^14,25–27^

While biomimetic bone scaffolds have been developed, they alone have not yet been able to robustly regenerate high-quality bone.^28,29^ The addition of stem cells and/or progenitor cells can significantly improve and accelerate bone healing.^30–33^ However, cell-based therapies also suffer from limitations tied to the source of cells, in vitro manipulation of cells, the rigorous regulatory approval process, and the associated high costs.^34–36^

In many situations (e.g. large defect repair, impaired tissue function), endogenous signal cues are not sufficient in type and/or amount to regenerate the damaged tissue, so the addition of exogenous signal cues is necessary for regeneration. The biomimetic tissue engineering strategy of incorporating signaling cues (both soluble and insoluble) aims to stimulate and accelerate the healing process. Delivering soluble signal molecules has been proven to promote bone repair,^35,37,38^ and several products containing growth factors have been used in orthopedic practice like spinal fusions^39,40^ and dental surgery.^41,42^ Bioactive signal molecules include small molecules,^43,44^ peptides/proteins,^38,45^ hormones,^46,47^ antibodies,^48,49^ and nucleic acids,^50,51^ which have been investigated for their ability to induce and accelerate bone regeneration. This review will discuss the types and mechanisms of the various bioactive signal molecules and general considerations in delivery system design. We will focus on the strategies and recent advances made in delivery systems as well as their potential in bone tissue engineering applications.

Besides soluble signal cues, insoluble physical cues (such as mechanical stimulation, ECM stiffness, and fluid flow) have also attracted significant attention due to their prospect of improving bone tissue formation.^52,53^ Such cues can significantly alter the cell shape, activity, and gene expression through the ECM–cell interactions and ultimately regulate cell migration, proliferation, and differentiation. They could directly facilitate the delivery of soluble signals to the site of bone defect, as well as improve the fixation and stability of the bone implant or tissue engineering constructs.^54,55^ A later section will discuss the biomimetic strategy for manipulating insoluble cues to facilitate bone regeneration.

## Fundamentals of drug delivery systems

In this section, the fundamental factors of drug delivery systems (DDSs) in bone tissue engineering will be discussed with emphasis on two aspects of design: the bioactive signal molecules and the delivery platform.

### Bioactive signal molecules in bone tissue engineering

Various biological signal molecules play important roles in regulating cellular activities and tissue development.^56^ Traditionally, the term “growth factor” refers to proteins or polypeptides capable of promoting tissue growth,^57^ but it does not accurately reflect other types and functions of bioactive signal molecules in certain situations. Besides the typical protein or polypeptide-based growth factors, many other types of signal molecules such as hormones and nucleic acids have also demonstrated great potential in promoting bone tissue regeneration. Each category of molecules possesses unique physiochemical properties and biological mechanisms, which require specific delivery system design strategies.

#### Growth factors

In this review the term “growth factor” refers to soluble, secreted signaling polypeptides or proteins, which can be synthesized by a wide variety of cells and play an important role in the regulation of cell proliferation, migration, differentiation, and ECM synthesis.^58^ Growth factors usually exhibit short-range diffusion through the ECM and act locally.^59^ A multitude of growth factors are involved in regulating the bone regeneration process and some of these growth factors (such as bone morphogenetic protein-2 (BMP-2),^60,61^ BMP-7,^38,62,63^ vascular endothelial growth factor (VEGF),^64,65^ and fibroblast growth factors-2 (FGF-2)^66,67^) have demonstrated great potential in numerous preclinical studies. Unfortunately, the promising results seen in animal models have yet to be translated successfully to human trials, mainly due to concerns over their side effects and safety. VEGF, for example, has a strong tendency to induce vascular permeability, which may lead to systemic hypotension and edema.^68,69^ Most of the complications concerning BMPs are related to heterotopic bone formation^70^ and BMP-2 is also known to increase risk of cancer development.^71^ The majority of growth factors that are currently used in clinical settings are delivered at a high enough dose to ensure the local concentration reaches therapeutic levels. Negative side effects associated with growth factors derive from poorly controlled drug release and supra-physiological level dosage. Therefore, there is a strong need to develop a delivery system for growth factors that allows an effective low dose to be delivered through precisely controlled release kinetics and tight localization in vivo.

#### Endocrine secretions

Endocrine secretion molecules (e.g. hormones) are a class of signaling molecules, produced by glands in multicellular organisms, transported by the circulatory system, and then targeting distant organs to regulate physiology such as tissue growth, function, and development.^72^ To reach their potential in promoting tissue regeneration, these molecules need to be delivered at specific time points and follow a certain release pattern.^73,74^ For example, parathyroid hormone (PTH) is a hormone that is crucial to regulating bone remodeling, where bone tissue is alternately resorbed and rebuilt over time.^75^ When given exogenously, pulsatile PTH administration promotes bone formation, whereas continuous PTH exposure results in bone resorption.^76^ Currently, PTH is the only FDA approved anabolic (i.e. bone building) agent for osteoporosis treatment in the US^77,78^ and its anabolic action has also been demonstrated to improve osseous healing.^79,80^ Although PTH has great potential as a regenerative agent to improve bone formation, its current administration via systemic injection is not suitable for localized defect regeneration. An engineered pulsatile system capable of delivering PTH to the local site to preserve its bioactivity and to induce the optimal anabolic action is highly desired.^81^

#### Nucleic acids

Nucleic acids alter cellular function and modulate the tissue regeneration process at the genetic level. DNAs and mRNAs encoding for growth and differential factors can enable protein expression for an extended period of time.^82^ For example, genes encoding for BMPs,^83–85^ FGF-2,^86^ insulin-like growth factors (IGFs),^87^ TGF-β,^88^ platelet-derived growth factor (PDGF),^89^ and VEGF^90^ have been shown to induce bone regeneration. In addition, the use of non-coding genes such as siRNA^91,92^ and miRNA,^50^ which regulate gene expression and cell activity, have recently emerged as novel therapeutic agents and also demonstrated great potential in bone repair. Owing to electrostatic repulsion, negatively charged nucleic acids (DNAs and RNAs) cannot easily cross the negatively charged cell membrane.^93^ The rapid degradation of some RNAs in vivo presents another challenge.^94^ For these reasons, gene vectors are usually used to protect and deliver genes in vivo. Viral and non-viral vectors have been used to deliver genes into the desired cells and each different vector has its advantages and disadvantages,^95,96^ which will be discussed in detail in the next section.

#### Other bioactive agents

A series of antibiotics and anti-inflammatory drugs have been also considered in combination with scaffolds^97^ for tissue engineering including bone repair.^43^ The aim is to combat bacterial and nonbacterial inflammation possibly introduced during and after the implantation of the tissue engineering construct.^98^ It is important to reduce inflammation in a wound to a level at which wound-healing processes can take place.^99,100^ It has been shown that sustained antibiotic release is effective in controlling infection at the bone defect site caused by debridement, and supports bone tissue healing.^15^ An antibiotic (vancomycin) loaded polycaprolactone (PCL) membrane has been developed as a DDS to control infection in a rabbit critical bone defect model.^101^ This antibiotic-delivery membrane was effective in reducing inflammatory cell infiltration, controlling bone infection, and improving bone repair.

Minerals such as calcium phosphonate (CaP) and hydroxyapatite have been widely used in a variety of orthopedic implants and scaffolds,^18,23,102^ and these minerals and the ions released have been demonstrated to promote pre-osteoblast proliferation and differentiation.^103,104^ Uniform and controlled deposition of minerals throughout the implants can be achieved through various methods. Simulated body fluid (SBF) incubation was originally developed to achieve mineral deposition onto scaffolds but this process was time-consuming, taking several weeks to form ideal mineral deposition.^105^ Subsequently, mineral electrodeposition has been developed and was able to rapidly generate mineralized CaP coating on the scaffold surface. This approach offers significant advantages over conventional SBF mineralization in that a high-quality mineral coating can be achieved within a short time (0.5–3 h typically) and the surface topography of the deposits can be tailored by controlling the electrochemical process parameters.^106^

### General considerations in drug delivery platform design

Although different types of signal molecules require specific delivery mechanisms, there are important universal considerations in the design of delivery platforms. Specific strategies and examples will be discussed in the next section.

#### Spatially controlled release within the regenerating tissue construct

The concentration and spatial distribution of signal molecules play a key role in tissue regeneration and development.^107^ The nearby cells sense the concentration gradient of signal molecules and respond in a concentration-dependent way.^108^ The drug concentration and spatial distribution near the delivery vehicle is the major determinant of the drug efficacy and effects. For example, numerous studies have shown that there is a threshold dose of BMP for in vivo bone induction and the amount of bone formation is largely dependent on the BMP dosage used. In a rat femur segmental defect model, a dose of 1.4 µg of BMP-2 did not result in union, whereas an 11 µg dose was sufficient for complete union.^109^ Therefore, it is crucial to deliver an effective amount of drug to the defect site. In general, the spatial distribution and local drug concentration are governed by both tissue physiology, which influences parameters such as diffusion and elimination rate, and the properties of the delivery system, which determine the drug release rate and dose. Insufficient control over the spatial distribution of the drug can lead to potential side effects and toxicity in non-target tissues. For example, the most recognized side effect related to BMP-2 use is ectopic bone formation, most likely due to BMP-2 leakage outside of the implant site.^110^ After spinal fusion surgery, ectopic bone formation occurs in the patients who were administrated with BMP-2 at a rate of nearly six times (70.1%) more than the control patients who were not administrated with BMP-2 (12.9%).^111^

#### Biomimetic temporally controlled release

The bone healing process involves multiple phases: the initial inflammatory phase, soft callus formation, mineralization, and bone remodeling. Multiple factors are involved in the different phases in specific temporal patterns.^112^ Biomimetic drug delivery strategies have been used to simultaneously or sequentially deliver multiple signals to mimic the natural healing process to synergistically enhance therapeutic effects and optimize the osteogenic outcome.^27,56^ It is known that natural osteogenesis is preceded by angiogenesis in the bone repair process. A combination of angiogenic (such as VEGF), cell recruiting (such as PDGF), and osteogenic (such as BMPs) growth factors has been designed and demonstrated a synergistic effect that is more beneficial to bone repair than any one growth factor delivered alone.^113,114^

Temporally controlled release is also critical for some drugs (e.g. endocrine secretion) that need to be delivered at specific time points and follow a certain pattern of distribution to be effective. Even given the same dose of a drug, distinct release patterns often lead to dramatic differences in the therapeutic outcome.^115^ Therefore, precise control over the temporal distribution of the drug is essential to achieving the desired therapeutic effect.^47^

#### Biocompatibility and safety issues

Biocompatibility and safety are required for DDSs and their tissue engineering applications. Many different materials can be used to fabricate the delivery vehicles, including synthetic polymers, natural polymers, and inorganic materials.^116^ The materials and their degradation products must be safe and biocompatible to not cause an excessive immune response.^117^ Typical synthetic polymers include poly(α-hydroxyester)s, polyanhydrides, polyorthoesters, poly(ethylene glycol) (PEG), and poly(vinyl alcohol) (PVA).^117,118^ The most commonly used poly(α-hydroxyester)s are homo- and copolymers of lactide and glycolide, because of their wide range of biodegradability and well-accepted biocompatibility.^119^ Some natural polymers such as fibrin, collagen, chitosan, alginate, and hyaluronic acid have also been widely used as these materials have an innate capacity to interact with cells and some undergo cell-mediated degradation.^120^ Silica-based inorganic materials have been investigated as drug carriers in preclinical studies and while they show low cytotoxicity, most of them are non-degradable in the human body.^121,122^

Additionally some responsive DDSs require external stimuli such as pH,^123,124^ temperature,^125,126^ light,^127,128^ ultrasound,^129,130^ electrical stimulation,^131^ and magnetic fields.^132^ The safety issues associated with application of these stimuli also require careful investigation. Safer and less-invasive stimuli are more likely to translate into clinical applications.

## Biomimetic strategies in drug delivery

**Fig. 1:**
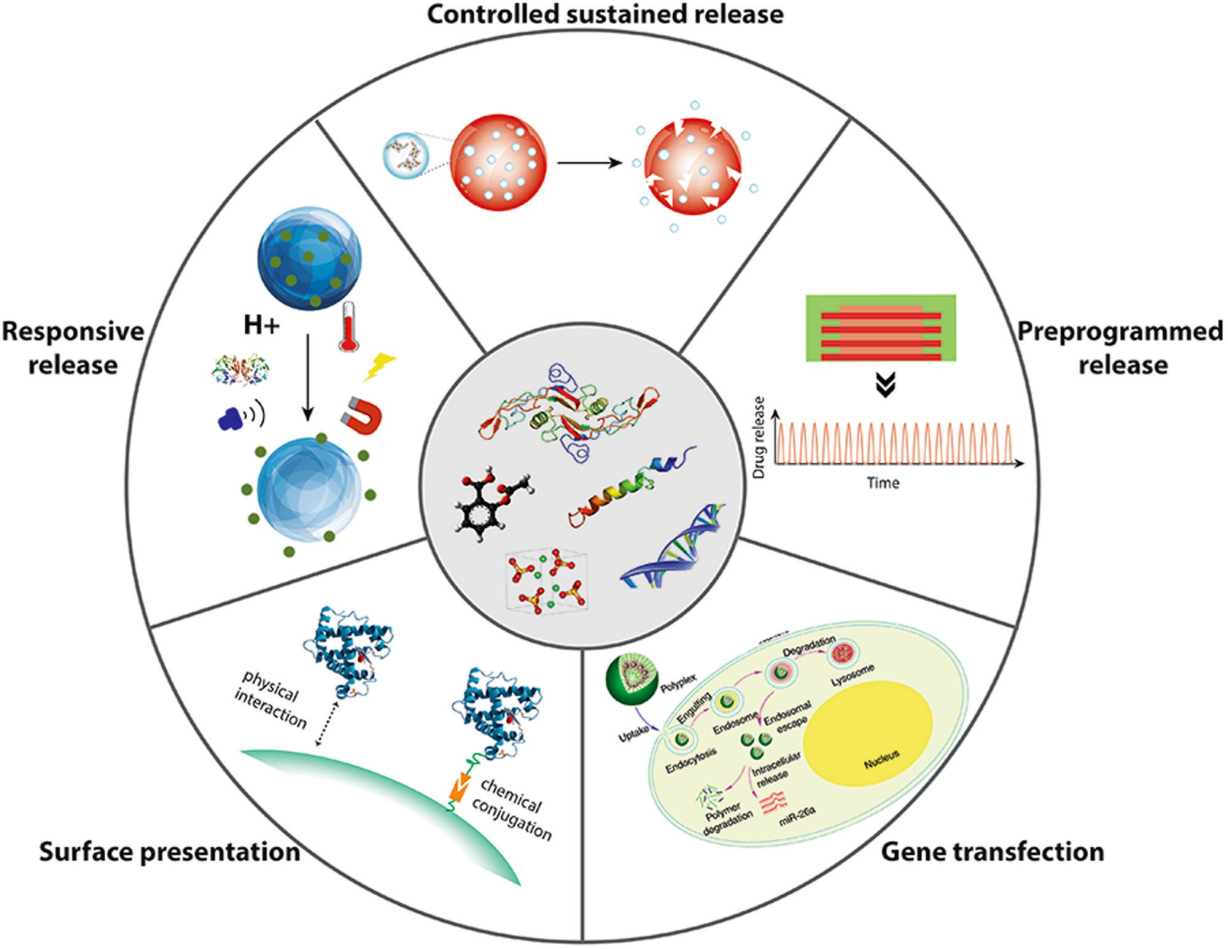
Various drug delivery strategies for signal molecules. Different types of signal molecules require different delivery systems to achieve optimal therapeutic effects. Delivery systems that have been developed and are currently used and/or are under investigation for bone tissue engineering applications include surface presentation, controlled sustained release, preprogrammed release, responsive release, and gene transfection. Copyright © 2016 by Nature Publishing Group, reprinted with permission of Nature Publishing Group, from Zhang et al.^50^

The importance and complexities of bioactive signal molecules in regulating cellular activities and bone healing suggests that DDSs mimicking the natural healing process are more likely to achieve advantageous therapeutic effects and thereby the desired bone regeneration outcome. Various methods and strategies have been exploited to enable spatiotemporal control over the drug release kinetics (Fig. 1 and Table 1).

**Table 1:** Summary of different types of bioactive signals and delivery strategies in bone tissue engineering applications

Types of bioactive signals	Delivery strategies and methods	Selected references
Small molecule	Surface adsorption, sustained release	^15, 43, 97^
Inorganics	Surface adsorption and deposition	^18, 23, 102, 106^
Growth factor	Surface presentation, controlled release	^38, 60, 147, 175^
Endocrines	Temporally controlled release	^47, 115, 164^
Nucleic acids	Intracellular and intranuclear delivery	^50, 83, 88, 89^

### Surface presentation

Various techniques have been explored to present drug molecules on the surface of the scaffold so that they are available for contact with cells migrating into the scaffold, acting as localized biological cues to regulate cell behavior.^133^ Surface presentation enables site-specific drug delivery and could reduce potential off-target side effects of the drugs. Physical adsorption and chemical conjugation are the two main methods for presenting drug molecules on the scaffold surface.

Physical adsorption usually relies on an interaction between the scaffold surface and the drug molecules, such as electrostatic interactions, hydrogen bonding, or hydrophobic interactions.^134,135^ The scaffold surface can be further modified to improve its affinity for drug molecules.^136^ For example, heparin has often been used to modify the scaffold surface chemically or physically to improve binding of the growth factors to the scaffold. There are many studies that report the controlled release of BMPs, PDGF, VEGF, and other growth factors in a heparin-modified scaffold.^138–140–141^ While there are certain preferred features, the passive adsorption approach could have limited control over drug retention and result in burst release or diminished bioactivity in some cases.^133^ Physiological conditions such as temperature, acidity, and mechanical movement could also interfere with the physical interactions, influencing the effectiveness of surface presentation.

**Fig. 2:**
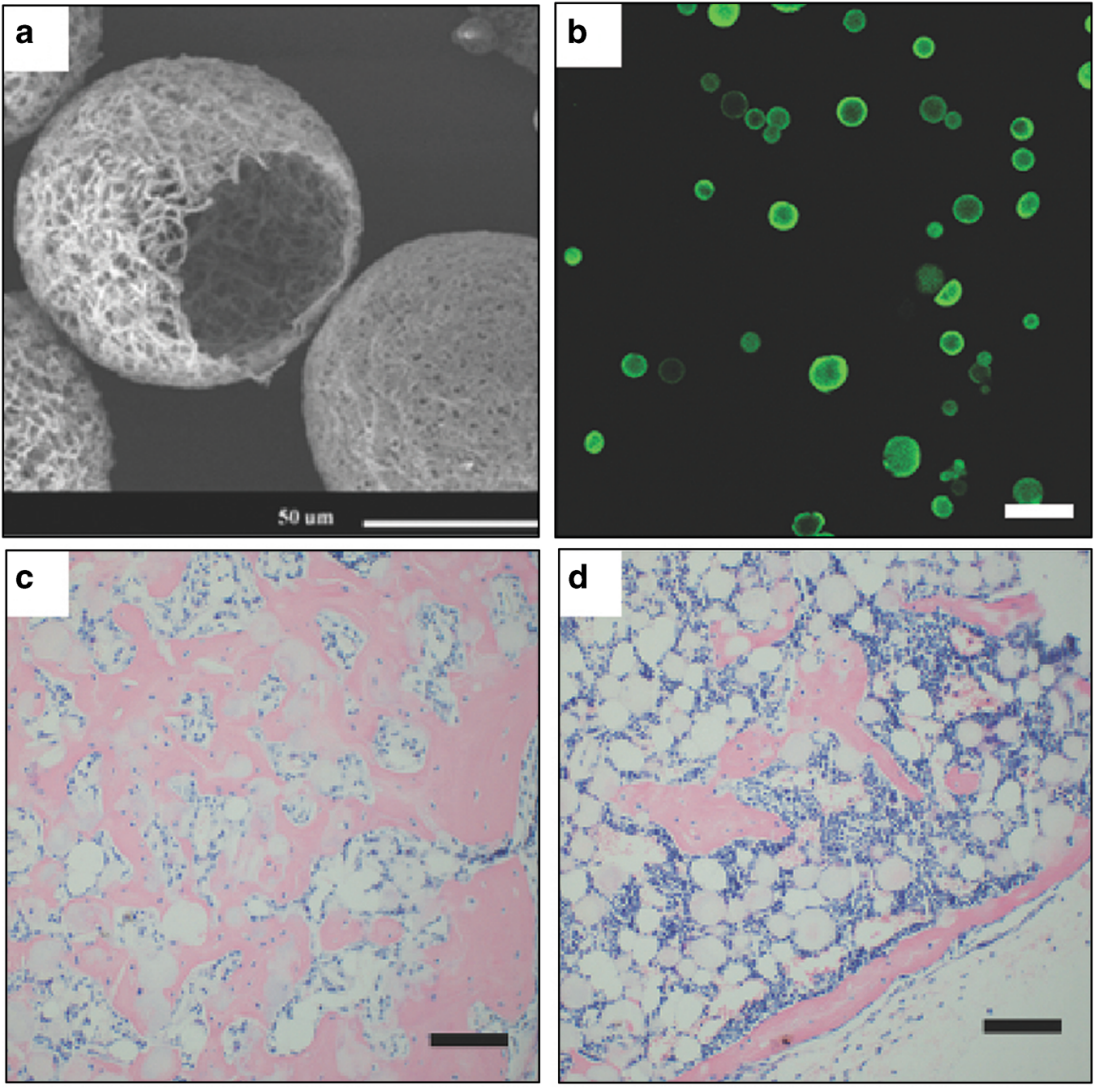
Surface modified nanofibrous microspheres with BMP-2-mimicking peptide induced stem cell osteogenesis and bone regeneration. **a** SEM images of surface modified nanofibrous microspheres. **b** A cross-sectional confocal image of nanofibrous microspheres after fluorescent labeling at the conjugation site, indicating that the microspheres’ surface has been functionalized with reactive groups for peptide conjugation. H&E analysis of BMP-2-mimic peptide conjugated microspheres (**c**) control microspheres (**d**) microspheres seeded with rabbit bone marrow stromal cells (BMSCs) after 5 weeks subcutaneous implantation. The BMP-2-mimicking peptide surface presentation strategy significantly induced osteogenic differentiation and promoted bone regeneration. Scale bars: 100 µm unless otherwise noted. Copyright © 2014 by John Wiley and Sons, reprinted with permission of John Wiley and Sons, from Zhang et al.^147^

Chemical conjugation, or covalent bonding, offers prolonged and more stable drug molecule presentation than the physical adsorption method. For this process, the scaffold surface needs to be activated with functional groups, which can then conjugate with drug molecules through the proper chemical reactions.^142^ However, in many cases, commonly used polymeric materials for bone tissue engineering are biodegradable polyesters, which lack reactive functional groups. In these instances, there are various methods to activate the scaffold surface via post-fabrication modification (such as plasma treatment, chemical etching, surface coating) but it should be noted that the activation treatment conditions need to be properly adjusted to maintain scaffold integrity.^143,144^ Another approach involves functionalizing the matrix materials or blending functional molecules with the main matrix materials prior to the scaffold fabrication. A primary concern is that the conjugation reaction may lead to a change in the conformation of the drug molecule, and result in loss of the bioactivity, especially for biologics. Therefore many drugs are pre-modified (e.g. conjugation to a PEG spacer)^145,146^ or drug mimics (growth factor peptide mimics)^147^ are utilized. Various bioconjugation reactions have been investigated, with reactions conducted in aqueous solution or under mild reaction conditions being particularly favorable. Amidation, esterification, and click reactions are among the most commonly used reactions.^148^ For example, BMP-2 mimicking peptide, P24, has been conjugated onto acrylic group-bearing PLLA nanofibrous (NF) scaffold via the thiol-ene click reaction. The scaffold decorated with BMP-mimicking peptide was able to retain its bioactivity and induce significant osteogenic differentiation of rabbit bone marrow-derived mesenchymal stem cells compared to a non-modified scaffold, inducing ectopic bone formation in nude mice (Fig. 2).^147^

### Controlled sustained release

Controlled sustained release of drug molecules is the most prominent drug delivery strategy in bone tissue engineering and aims to provide the desired drug concentration at the local regeneration site. Physical encapsulation of the drugs into polymeric materials is the most commonly used method and the release kinetics can be controlled by the matrix degradation and drug diffusion rate.^149^ The drugs can be either encapsulated inside the scaffold matrix^150^ or in separate delivery vehicles such as micro/nanoparticles,^151^ or liposomes.^152^

Direct loading of the drugs into the scaffold matrix is the simplest way to achieve sustained drug release and many techniques have been developed including solvent casting, in situ polymerization, phase separation, electrospinning, gas foaming, and more.^154–155^ The major challenge with this strategy is to protect the bioactivity of the drugs from the harsh scaffold fabrication process. Most growth factors and other types of biologics cannot be dispersed directly in polymer solution as the solvent can temper their bioactivity. Another drawback of this strategy is the lack of control over the release kinetics. For example, growth factors have been electrospun into NF scaffolds but they usually aggregate on the outer surface, resulting in a burst release.^133^

**Fig. 3:**
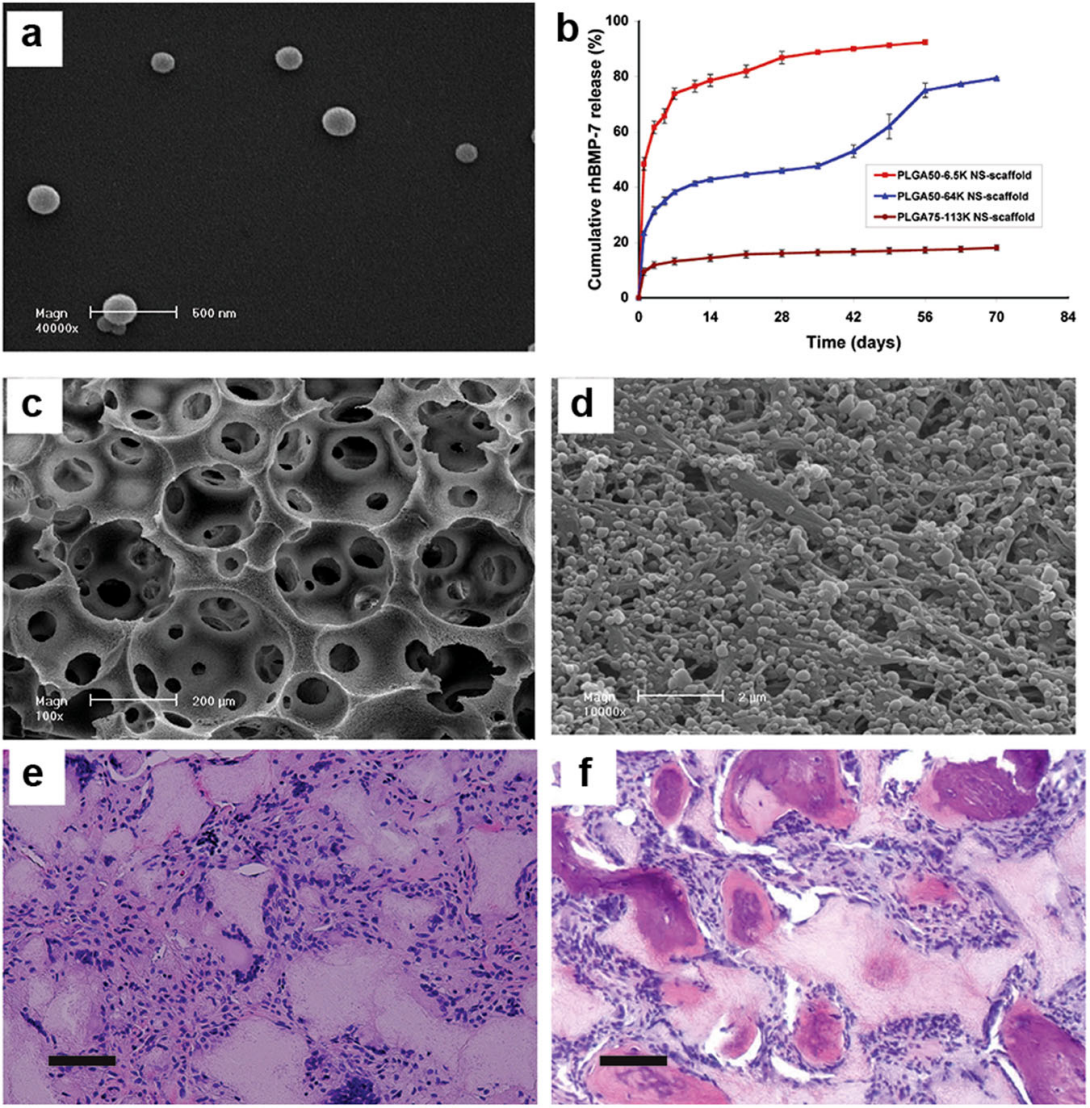
BMP-7-releasing nanofibrous scaffold. **a** SEM images of BMP-7-encapsulated PLGA nanospheres prepared via double emulsion method. **b** In vitro BMP-7 release profiles. The release kinetics were modulated by tailoring the chemical and physical properties of the polymer matrix. SEM micrographs of PLGA nanospheres-immobilized on porous NF scaffolds at (**c**) lower magnification, ×100 and (**d**) higher magnification, ×10 000. The drug-releasing nanospheres were immobilized onto the internal surface of the scaffold pores. H&E staining of tissue formation in the BMP-7 absorbed scaffold (**e**) and the BMP-7 controlled releasing scaffold (**f**) in mouse subcutaneous implantation model, showing that significant bone was regenerated in the BMP-7 controlled release group. Scale bar: 100 µm in (**e** and **f**). Copyright © 2006 by Elsevier, reprinted with permission of Elsevier, from Wei et al.^38^

Encapsulating the drugs in separate delivery vehicles instead of directly within the scaffold matrix is a promising alternative strategy. Micro/nanospheres have long been used for drug encapsulation and various methods have been investigated to retain the drug bioactivity and achieve controlled release kinetics.^38^ Additionally, a solvent annealing technique has been developed to immobilize the drug-loaded microspheres on the scaffold surface.^156^ This method enables single or multiple drugs to be released in a spatially and temporally controlled fashion throughout the scaffold and enables the drug release profiles to be individually designed without altering the scaffold structure (Fig. 3). BMP-7-loaded microspheres have been immobilized onto cell-free NF scaffolds, which were tested in subcutaneous implantation in rats.^38^ BMP-7 was released from the scaffold-bound microspheres in a controlled fashion with high bioactivity and induced bone formation within the scaffold. In contrast, the scaffolds with BMP-7 adsorbed were unable to induce osteogenesis, owing to the loss of bioactivity and short release duration of the BMP-7. In another study, such BMP-loaded microspheres mixed with an injectable scaffold and stem cells of apical papilla induced ectopic mineralized dentin formation.^157^

Drug molecules can also be incorporated into liposomes; however, they are relatively unstable in a physiological environment, which results in short release duration.^158,159^ Sustained drug release can also be achieved using porous inorganic materials but safety and degradation issues limit their potential for translation into clinical applications.^160^

### Preprogrammed release

While sustained delivery is a simple form of preprogrammed delivery, more sophisticated preprogrammed delivery systems are designed to utilize the spatiotemporal sensitivity of a patient to drugs to achieve optimal therapeutic effects.^161,162^ A common approach to achieve preprogrammed drug delivery involves multi-compartment constructs where drugs are pre-loaded into different compartments that have different release kinetics.^45,163^ Bulk- and surface-eroding polymers have been used as matrix materials for the compartments and can be engineered to achieve the desired release kinetics. Another strategy takes advantage of the development of nano/microfabrication techniques and functional materials to make drug delivery chips and responsive vehicles, where the drug release can be either programmed in the chip or triggered by remote stimuli.^164^

Preprogrammed DDSs are used to facilitate sequential release of multiple drugs to enhance bone regeneration. Instead of delivering a single molecule at a high dose, the safety and efficiency of the delivery system can be improved by delivering multiple signal molecules at relatively low doses in a sequential manner. DDSs loaded with multiple osteogenic and/or angiogenic factors have shown improved osteogenic outcomes.^62,163,165,166^

**Fig. 4:**
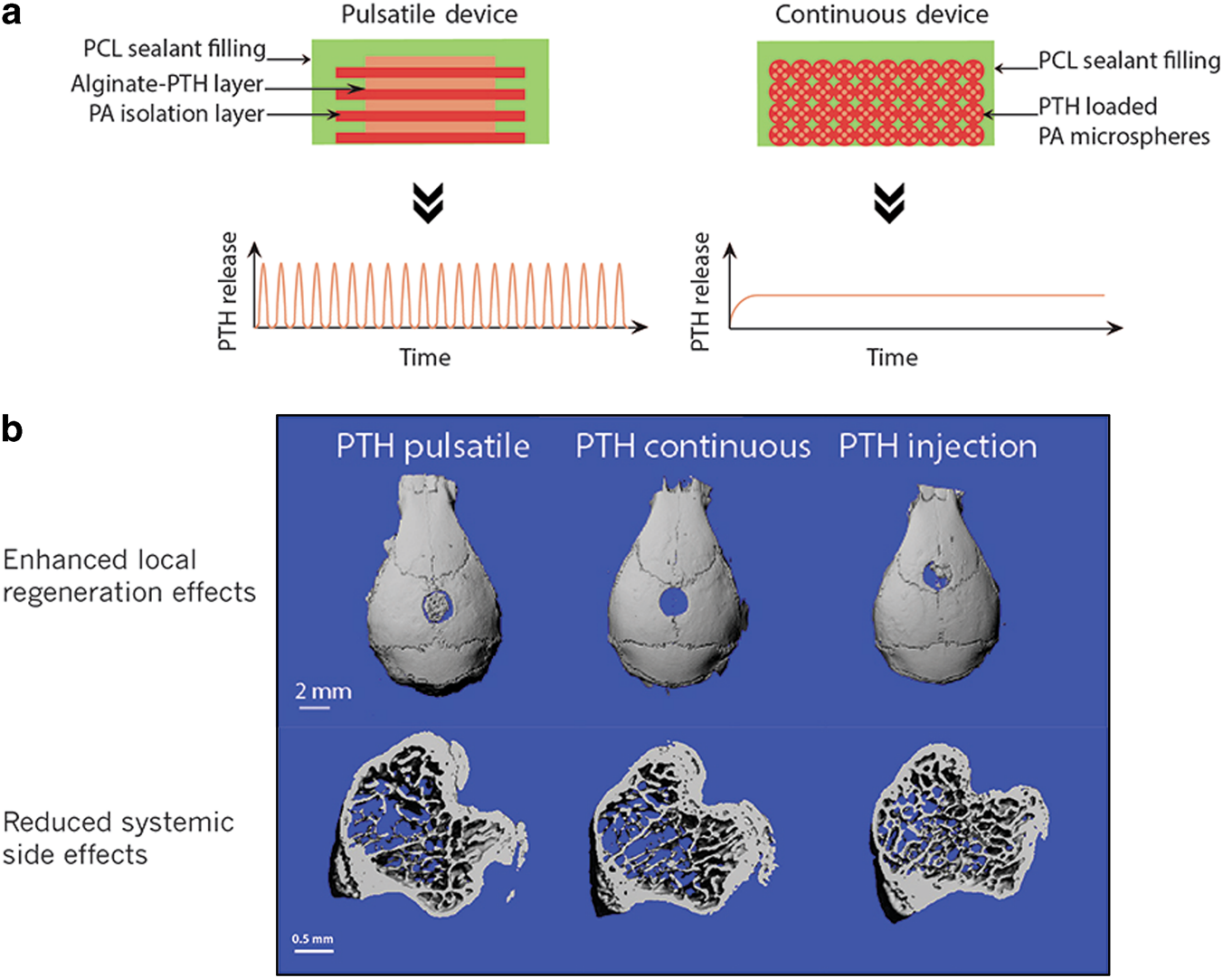
Preprogrammed PTH delivery system for local bone defect repair. **a** Schematic illustration of PTH delivery device (pulsatile and continuous). Two types of devices made with the same biodegradable materials and loaded with the same amount of PTH but delivered PTH in distinct manners, pulsatile or continuous, for 21 days. **b** Representative µCT characterization of mouse calvarial defect (top panel) and intact tibiae (bottom panel) in response to the different PTH delivery systems in vivo. PTH delivery device (pulsatile or continuous) was implanted in the calvarial defect locally. A subset of control mice received standard PTH subcutaneous injection (40 µg/kg/day) for 21 days. The PTH pulsatile device significantly enhanced the PTH anabolic effects in regenerating bone compared to the standard PTH injection, whereas the PTH continuous device resulted in bone resorption; PTH local delivery (pulsatile or continuous) resulted in negligible systemic effect compared to the PTH injection treatment. Copyright © 2016 by Elsevier, reprinted with permission of Elsevier, from Dang et al.^47^

For some drugs, different release patterns for the same drug can lead to distinct therapeutic outcomes, so achieving the specific drug release pattern is essential to achieve the desired therapeutic effects. As previously discussed, the anabolic or catabolic action of PTH highly depends on its delivery pattern. Intermittent PTH administration (such as daily injection) improves bone microarchitecture, mineral density, and strength, whereas continuous exposure of PTH leads to bone resorption.^75–77^ Our laboratory recently developed a novel preprogrammed delivery device capable of long-term (e.g. 3 weeks) pulsatile release (Fig. 4).^47,115^ This device enabled local pulsatile PTH release to the bone defect site. Our results showed that the local pulsatile PTH release significantly improved the bone regeneration, achieving more robust bone repair with negligible side effects compared to the standard 3-week daily systemic injections.^47^

### Responsive release

Responsive delivery systems that release drugs in response to local physiological signals or external stimuli have recently received increasing attention.^167^ Such on-demand drug release could reduce side effects caused by excess drug dosing found in conventional administration.^168,169–170^ Commonly used stimuli include pH, temperature, exposure to an electric or magnetic field, ultrasound, light irradiation, and biomolecules. These responsive systems involve “smart” responsive materials, so that the materials undergo considerable changes in response to the stimuli and thereby change the drug release kinetics (e.g. release rate, dosing, and duration).

Responsive delivery systems have been used in systemic treatments, including many common cancer therapies^168,171,172^ but with limited success in tissue engineering applications. The regeneration process often requires long-term drug release, while most of the responsive systems suffer from the short release duration and irreversible responsive release.^173^ Additionally, the insufficient biocompatibility and biodegradability of the functional materials as well as the safety concerns associated with the external stimuli limit their broad application in tissue engineering.

One promising class of responsive delivery systems for tissue engineering is the biomolecule-sensitive system.^174^ This kind of bio-inspired system is triggered by the local environment and would not need external stimuli. A protease cleavage-based triggering mechanism has been developed to initiate local drug release. A metalloproteinase (MMP) is a protease that is upregulated in angiogenesis and cell invasion. An MMP-cleavable crosslinker has been incorporated into a PEG-based hydrogel for local rhBMP-2 delivery to the site of bone defects.^175^ Prior to MMP exposure, BMP-2 remained as a precipitate within the PEG matrix and was not released over a period up to 5 days. After implantation in the bone defect, cells migrate and invade the hydrogel matrix resulting in MMP secretion and the degradation of the MMP-sensitive PEG network. This causes BMP-2 dissolution and release, leading to efficient bone regeneration. Similarly, cell-mediated release of VEGF has demonstrated local and controlled induction of angiogenesis.^176^

### Gene transfection

The goal of gene therapy is to deliver genes to the bone defect area so that they can regulate the expression of biomolecules (such as proteins) and cell activity to enhance proliferation and/or osteogenic differentiation. Gene transfection is often mediated by either a viral vector^96,177^(such as retrovirus, adenovirus, adeno-associated virus, herpesvirus) or a non-viral vector^178,179^ (such as polycations or liposomes). Viral vectors have shown high gene transfection efficiency; however, associated safety issues, immune response, and side effects are of serious concern and greatly limit the translation of this approach into clinical use. The main advantage of non-viral vectors over viral vectors is their superior safety with additional benefits including ease of fabrication and scale up. Liposome-based transfection vectors including Lipofectamine (a commercial non-viral vector) have been used to transport genes into cells in vitro but most liposomes exhibit low stability in the physiological environment.^180^ Polymeric non-viral vectors, including polyethyleneimine, chitosan, and other polycation-based vectors often suffer from low efficiency and considerable cytotoxicity.^181,182^ Moreover, both viral and non-viral transfection systems only allow for one-time or short-term delivery, whereas sustained release is typically required for bone regeneration. For this reason, there have been a limited number of gene therapies capable of in vivo bone regeneration reported.^183^

**Fig. 5:**
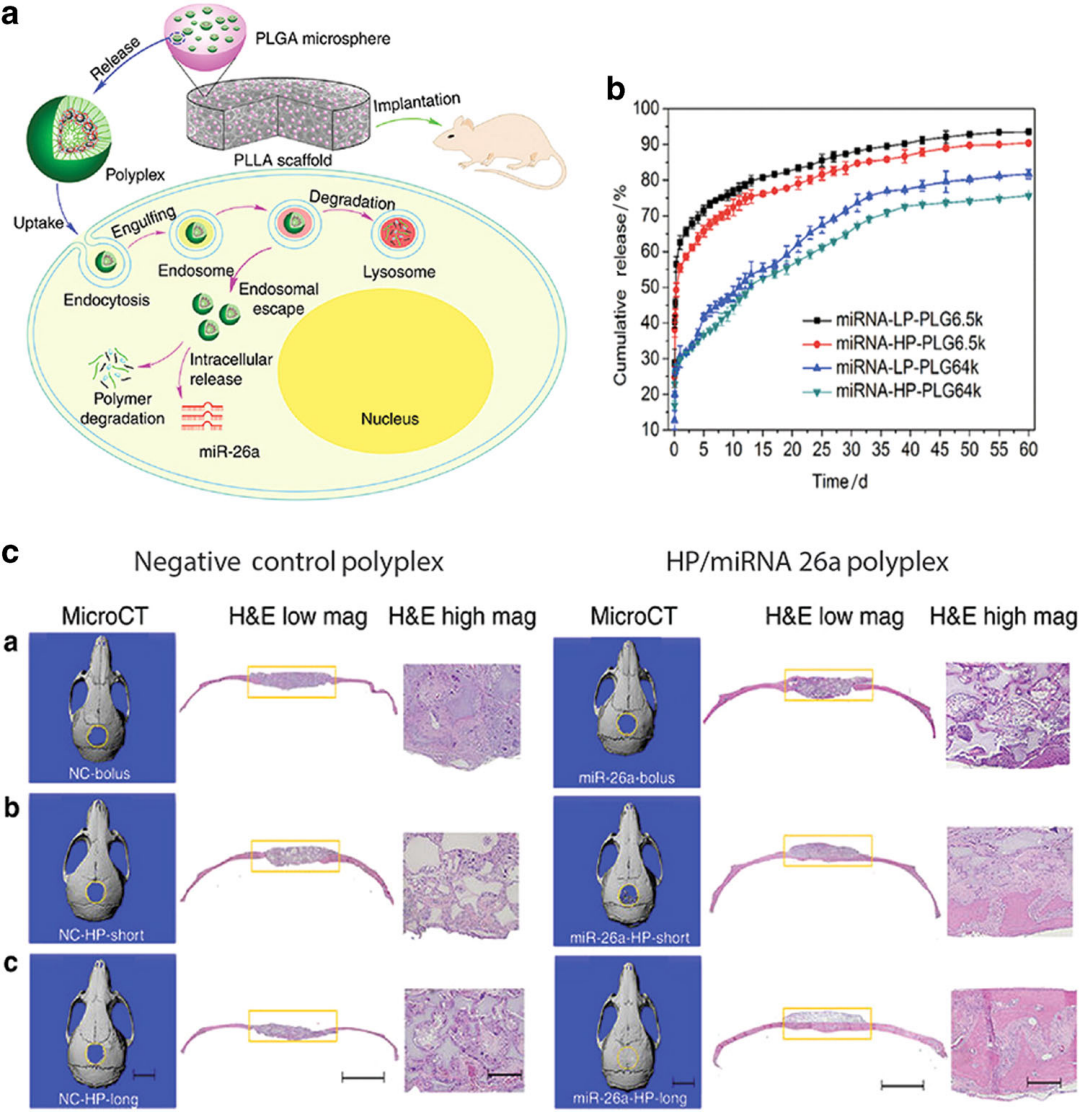
Two-stage delivery of miRNA-26a from scaffold repaired critical bone defects. **a** Schematic illustration of the two-stage miRNA delivery system. The miRNA and gene vector formed polyplexes in water, which were encapsulated into PLGA microspheres, followed by immobilization onto the PLLA scaffold. The implanted scaffold filled in the mouse calvarial defect. The PLGA microspheres released miRNA–vector complex or polyplexes in vivo and the polyplexes were taken into cells through endocytosis. Once inside the cell, the intracellular release of miRNA regulated subsequent gene expression. **b** Release profiles of miRNA from different PLGA (6.5 or 64 kDa) microspheres containing different gene/vector complexes (LP or HP). The release durations (short and long) were controlled by tuning the polymer matrix. **c** Representative µCT and H&E analysis of scaffolds in the mouse critical defect model. (a) Cell-free scaffold with the NC polyplexes (miR-26a-bolus or NC-bolus) or miRNA-26a/HP vector polyplexes bolus; (b) cell-free scaffold with short-term releasing PLGA microspheres that contains the NC polyplexes (miR-26a-bolus or NC-bolus) or miRNA-26a/HP vector polyplexes; (c) cell-free scaffold with long-term releasing PLGA microspheres that contains the NC polyplexes (miR-26a-bolus or NC-bolus) or miRNA-26a/HP vector polyplexes. Results showed that the two-stage delivery of miRNA-26a repaired the critical calvarial bone defect in vivo and the long-term sustained release was much more advantageous than the short-term release. Scale bars, 5 mm (in μCT images), 2.0 mm (in H&E images at right), 200 mm (in higher-mag H&E images at far right). Copyright © 2016 by Nature Publishing Group, reprinted with permission of Nature Publishing Group, from Zhang et al.^50^

New approaches are being actively pursued to overcome current limitations in gene delivery for bone tissue engineering. Recently a water-soluble hyperbranched polymeric vector was developed that can incorporate the desired genes when it assembles into a stable nanoshell structure.^50^ This unique vector–gene complex, or polyplex, provides excellent stability and the polyplexes remained intact even after harsh sonication. Polyplexes loaded with miRNA-26a have been encapsulated in biodegradable polymer microspheres (Fig. 5)^50^ to achieve long-term controlled miRNA-26a release. This delivery system enabled two-stage control over the gene release: sustained release of the polyplexes from the microspheres and efficient gene transfection into cells by the polyplexes. Microspheres loaded with the polyplexes were immobilized within the scaffold to provide spatially controlled distribution to prevent undesired off-target side effects. This study showed that the delivered miRNA-26a targets Gsk-3β to increase osteoblast activity, resulting in the regeneration of critical-sized calvarial bone defects in both healthy and osteoporotic mice.

## Insoluble cues

Insoluble cues can regulate the function of bone-related cells, influence the property and structure of ECM, and eventually affect mineralization and bone regeneration.^184,185^ Bone-related cells, including osteoclasts, osteoblasts, as well as osteocytes and precursor cells or bone lining cells, respond to physical cues and biomechanical stimulations from the physiological microenvironment.^186,187^ For example, matrix modulus can have a dominant effect on the fate of naïve mesenchymal stem cells. A matrix of a higher elastic modulus (25 kPa and higher) induces the osteogenic differentiation, while matrices of lower elastic moduli (such as 0.1–1 or 8–17 kPa) lead to neurogenic differentiation or myogenic differentiation of these mesenchymal stem cells.^52^ These findings indicate insoluble cues are critically important in bone regeneration and can be incorporated into scaffold design.^188^ For example, scaffolds with mechanical properties (such as stress, modulus, and viscoelasticity) similar to the natural bone ECM have been developed. These mechanomimetic scaffolds can regulate the fate of marrow stromal cells (MSCs) and are reported to regenerate tissue with similar structure and performance to native tissue.^189,190^

Various mechanical loadings have also been applied to cells and tissue engineering constructs in vitro to mimic the natural microenvironment and have demonstrated significant effects on cell behavior. For example, MSCs were cultured in a flow perfusion system and the osteogenic differentiation of MSCs is enhanced with the presence of fluid shear stress (FSS).^191^ The mineralized matrix deposition is also increased by increasing FSS.^192–194^ Further studies have revealed that dynamic fluid flow with higher peak shear stress amplitudes, faster oscillating frequencies, and longer loading durations provide the best conditions for promoting bone formation.^195^ It has been shown that tissues with higher modulus increase the accumulation rate of d-aspartic acid, which is positively correlated with the half-life of collagen^196^ and thereby could influence the collagen properties.^197^ Besides, it has been found that FSS can affect both the transition of amorphous calcium phosphate (ACP) precursor towards hydroxyapatite and the crystal structure and orientation.^198,199^ Recently, researchers demonstrated that biomechanical stimuli regulate collagen assembly and the mineralization process. About 1.0–1.5 Pa FSS has a positive effect on the biomineralization process, as evidenced by the enhanced degree of collagen self-assembly and the accelerated speed of ACP formation and transition. Under the optimal FSS condition, ACPs of well-controlled size have been formed with the minerals uniformly dispersed inside of collagen fibrils, resulting in intrafibrillar mineralization.^200,201^ Of note, biomechanical stimulation can also affect the degradation and release properties of PLA and PLGA polymers.^202–204^

## Conclusions and future outlook

Significant progress has been made both in the understanding of the molecular mechanisms in tissue development and regeneration and in the design of biomimetic biomaterials. Various biomimetic DDSs have been developed to mimic the natural healing or development process and provide spatiotemporally controlled drug release. The integration of DDSs with bone implants or scaffolds could lead to advanced tissue engineering therapy for bone defect repair. Biomimetic DDSs can also serve as a novel platform to study the basic biology of how signal molecules manipulate cell activity and tissue development.

Although drug delivery-based approaches have been utilized in several orthopedic and craniomaxillofacial repairs, the safety and cost-effectiveness issues are driving the research towards the development of optimal DDSs that allow drug dose reduction and precise localization. A variety of biological and engineering challenges remain to be addressed. First, further qualitative and quantitative understandings of the complex interactions and cooperative signaling between the signal molecules, microenvironment, and physiology could provide guidance to achieve precise spatiotemporal control and prediction of drug release and distribution. Second, development of more sophisticated delivery platforms should rely on multidisciplinary approaches that are based on biological principles and combine nanotechnology, advanced fabrication, and functional materials to deliver the required signals to the desired cells at the right time. In addition, emerging drug delivery technologies and platforms, such as responsive materials, remotely controlled delivery devices, and microchip-based devices have shown beneficial potential, but further research is needed to investigate how to incorporate these innovations into bone tissue engineering applications and future clinical practice.

## Electronic supplementary material

Figure reuse permission-1

Figure reuse permission-2

Figure reuse permission-3

Figure reuse permission-4
