# Supplementary material for: Biomimetic delivery of signals for bone tissue engineering
Source: Bone Res. 2018 Aug 29;6:25. doi: 10.1038/s41413-018-0025-8 (PMC6115422; doi:10.1038/s41413-018-0025-8)
Supplement: Supplementary file 1 — Figure reuse permission-1 [file 41413_2018_25_MOESM1_ESM.pdf]

Copyright Clearance Center RightsLink®

Home | Account Info | Help

nature publishing group

Title: Cell-free 3D scaffold with two-stage delivery of miRNA-26a to regenerate critical-sized bone defects

Author: Xiaojin Zhang, Yan Li, Y. Eugene Chen, Jihua Chen, Peter X. Ma

Publication: Nature Communications

Publisher: Nature Publishing Group

Date: Jan 14, 2016

Copyright © 2016, Rights Managed by Nature Publishing Group

Logged in as:
ming dang
Account #:
3000679349

LOGOUT

Author Use

Authors of NPG articles do not require permission to use content from their article in most cases as stated in the author's guidelines.

Authors wishing to use their article for commercial purposes must request permission in the normal way.

For further questions, please contact NPG's permissions department: permissions@nature.com

BACK | CLOSE WINDOW

For commercial reprints of this content, please select the Order Commercial Reprints link located beside the Rights and Permissions link on the Nature Publishing Group Web site.

Copyright © 2017 Copyright Clearance Center, Inc. All Rights Reserved. Privacy statement. Terms and Conditions.
Comments? We would like to hear from you. E-mail us at customercare@copyright.com
